# Long-term brain structural magnetic resonance imaging and cognitive functioning in children treated for acute lymphoblastic leukemia with high-dose methotrexate chemotherapy alone or combined with CNS radiotherapy at reduced total dose to 12 Gy

**DOI:** 10.1007/s00234-016-1777-8

**Published:** 2017-01-10

**Authors:** Olga Zając-Spychała, Mikołaj A. Pawlak, Katarzyna Karmelita-Katulska, Jakub Pilarczyk, Katarzyna Derwich, Jacek Wachowiak

**Affiliations:** 1 Department of Pediatric Oncology, Hematology and Transplantology, Poznan University of Medical Sciences, Szpitalna Str. 27/33, 60-572 Poznan, Poland; 2 Department of Neurology and Cerebrovascular Disorders, Poznan University of Medical Sciences, Poznan, Poland; 3 Department of Neuroradiology, Poznan University of Medical Sciences, Poznan, Poland

**Keywords:** Acute lymphoblastic leukemia, Brain, CNS prophylaxis, Cognitive functioning

## Abstract

**Introduction:**

The aim of this study was to assess the long-term side effects of central nervous system prophylaxis (high-dose chemotherapy alone vs chemotherapy and CNS radiotherapy) according to the ALL IC-BFM 2002.

**Methods:**

Thirty-tree children aged 6.7–19.9 years have been studied. The control group consisted of 12 children newly diagnosed with acute lymphoblastic leukemia. We assessed subcortical gray matter volume using automatic MRI segmentation and cognitive performance to identify differences between two therapeutic schemes and patients prior to treatment.

**Results:**

Patients treated with chemotherapy and CNS radiotherapy had smaller hippocampi than two other subgroups and lower IQ score than patients treated with chemotherapy alone. Both treated groups, whether with chemotherapy only or in combination with CNS radiotherapy, had significantly lower volumes of caudate nucleus and performed significantly worse on measures of verbal fluency in comparison with patients prior to treatment. There were no differences in the mean volumes of total white matter, total gray matter, thalamus, putamen, and amygdala between the studied groups.

**Conclusion:**

In all children treated according to the ALL IC-BFM 2002 with high-dose chemotherapy, both decreased volume of selected subcortical structures and cognitive impairment was observed, especially in children who received chemotherapy in combination with reduced dose CNS radiotherapy. In all children treated according to the ALL IC-BFM 2002 with high-dose chemotherapy, both decreased volume of selected subcortical structures and cognitive impairment were observed, especially in children who received chemotherapy in combination with CNS radiotherapy.

## Introduction

Acute lymphoblastic leukemia (ALL) is the most common malignancy in children [1–3]. Treatment of ALL in children based on the ALL IC-BFM 2002 protocol (ALLIC 2002) is composed of multi-drug chemotherapy adjusted for three risk subgroups (standard, intermediate, and high) administered repeatedly for 24 months and central nervous system (CNS) radiotherapy given to a particular group of patients. Even though only 4% of the cases have CNS involvement at diagnosis [4], CNS-directed prophylaxis is a mandatory part of ALL therapy in all cases due to high risk of CNS relapse [3, 5, 6]. According to ALLIC 2002, CNS prophylaxis—depending on risk group and immunophenotype of leukemia—consists of systemic chemotherapy with intravenous methotrexate in high or medium doses, intrathecal methotrexate alone, or in combination with cytarabine and prednisone and CNS radiotherapy at total dose of 12 Gy which is used only in patients stratified into high-risk group and T-cell ALL.

Prior reports on cognitive assessment following cranial radiotherapy at an 18-Gy dose or higher demonstrated that such approach may lead to diminished intelligence quotient, memory, attention, and processing speed deficits [7–9]. However, the impact of 12-Gy dose radiotherapy on cognitive functioning remains unknown. Some authors showed adverse effects of methotrexate, both intrathecal [10] and intravenous [11], on long-term neuropsychological outcome in ALL survivors, while some others did not observe such consequences [6]. There were reports indicating leukoencephalopathy defined as white matter (WM) hyperintensities on T2-weighted images related to methotrexate used in combination with CNS radiotherapy [12]. Currently, there is very limited evidence regarding impact of the therapy on gray matter (GM) structures and its relationship to cognitive performance in ALL survivors.

However, even though there is very limited data on GM impairment and data on association of structural imaging features with cognitive processes in children survived from ALL is still lacking, the role of basal ganglia is well known. Basal ganglia are clusters of GM within the basal part of the forebrain. Due to the complex structural and functional connections of basal ganglia with widespread regions of the cortex, especially with associative prefrontal cortex, they contribute to many functions. These are selecting and enabling various cognitive, executive, or emotional programs that are stored in other cortical areas and involvement in certain types of learning and in the enabling of practiced motor acts as well as in gating the initiation of voluntary movements by modulating motor programs stored in the motor cortex. However, despite the fact that there are some papers concerning the correlation between cortex volume and cognitive performance in patients who were treated with chemoradiotherapy, there is very little data regarding the impact of subcortical structures on functioning. Nevertheless, clinical studies support observations that dysfunction in individual basal ganglia loops with the cerebral cortex may impact neurocognitive performance [13].

The aim of this cross-sectional study was to evaluate the impact of high-dose methotrexate and 12-Gy radiation therapy on total WM, total GM and subcortical structures volumes, and cognitive performance in ALL children. We hypothesized that reduced volume of the subcortical structures would be associated with treatment effect of chemotherapy and radiotherapy. We also hypothesized that observed cognitive deficits would be associated with volumetric measurements of structures involved in impaired functions.

## Patients and methods

### Study design and patient population

This study was approved by the local Institutional Review Board. Information about the trial was presented to potential participants and their parents. Written informed consent was obtained from all participants.

**Fig. 1:**
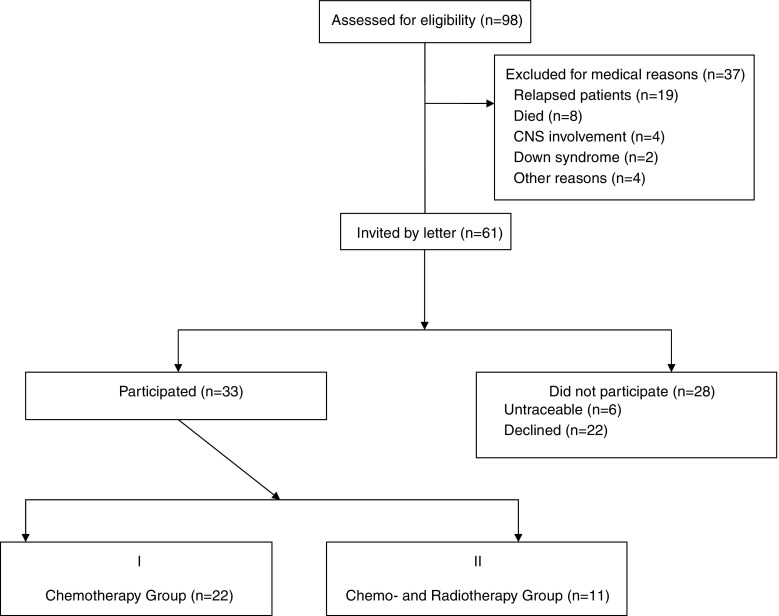
Flow diagram of ALL survivor selection

We retrospectively identified 98 children cured from ALL and treated according to ALLIC 2002 between December 2002 and December 2007. Thirty-seven patients were not eligible for the follow-up study (19 patients relapsed; 8 patients died because of treatment-related complications; 4 patients had CNS involvement at ALL diagnosis; 2 had Down syndrome; 4 were not eligible for other causes, i.e., mental retardation in 3 cases and Gypsy origin in 1 case). The other 61 children were invited to participate in the study. From this group, 28 children did not participate (6 patients were untraceable and 22 declined to participate). The other 33 families agreed to participate in the cross-sectional prospective study. Out of the 33 children who were tested, 22 children (11 female) of median age of 12.1 years (interquartile range (IQR) 9.5–14.4, range 7.6–19.9) had previously been treated according to standard or intermediate-risk group of ALLIC 2002 with chemotherapy alone (group I). The other 11 children (5 female) of median age of 11.6 years (IQR 8.3–13.2, range 6.7–18.6) had previously been treated according to the high-risk group with chemotherapy and cranial radiotherapy (group II). Flow diagram of the patients’ selection is shown in Fig. 1. The median time from the therapy completion to the evaluation was 50 months (range of 30–75 months). The control group consisted of 12 children (5 female) of median age of 11.8 years (IQR 9.2–13.7, range 6.4–17.5) newly diagnosed with ALL without CNS involvement and tested before the chemotherapy and corticosteroids started (group III). Summary of patients in each studied group is presented in Table 1.

**Table 1:** Characteristics of patients

	Group I (chemotherapy only)	Group II (radiochemotherapy)	Group III (control group)	*p*
Number (male/female)	22 (11:11)	11 (6:5)	12 (7:5)	–
Range of age (median; IQR)	7.6–19.9 (12.1; 9.5–14.4) years	6.7–18.6 (11.6; 8.3–13.2) years	6.4–17.5 (11.8; 9.2–13.7) years	0.480
Time from therapy ended (median)	31.5–71.4 (50.7) months	30.0–75.2 (57.0) months	–	0.689
Range of age at diagnosis (median; IQR)	2.7–13.9 (5.2; 4.3–8.2) years	2.8–12.9 (4.9; 3.9–8.8) years	–	0.633

### Therapeutic regimen

The ALLIC 2002 regimen was based on multi-drug chemotherapy consisting of induction therapy, consolidation, reinduction, and maintenance therapy. The treatment intensity had been stratified into one of three risk subgroups: standard, intermediate, or high. Children had been stratified into the high-risk group if one of the following factors occurred: presence of t(9;22) (BCR/ABL+) or t(4;11) (MLL/AF4+), poor corticosteroid response (peripheral blasts count of >1 × 10^9^/L on day 8 of initial treatment), or lack of hematological remission on day 33 (>5% of blasts in bone marrow). The remaining children had been classified as standard or intermediate risk. For all risk groups, induction therapy had included prednisone, vincristine, daunorubicin, asparaginase, cyclophosphamide, cytarabine, mercaptopurine, and intrathecal methotrexate. The consolidation treatment for standard and intermediate-risk group had included intermediate doses of methotrexate (MD-MTX; 2 g/m^2^) with intrathecal methotrexate or high doses of methotrexate (HD-MTX; 5 g/m^2^), cytarabine, and vincristine. High-risk group had received vindesine, asparaginase, cyclophosphamide or ifosfamide, and daunorubicin, etoposide with triple intrathecal therapy (methotrexate, cytarabine, prednisone). Reinduction therapy had been the same for all patients and included vincristine, doxorubicin, asparaginase, cyclophosphamide, cytarabine, thioguanine, and intrathecal methotrexate. High-risk patients had received 12-Gy whole-brain radiotherapy as a prophylaxis of CNS prior to standard maintenance therapy: mercaptopurine and methotrexate.

### Magnetic resonance imaging—acquisition and analysis methods

**Fig. 2:**
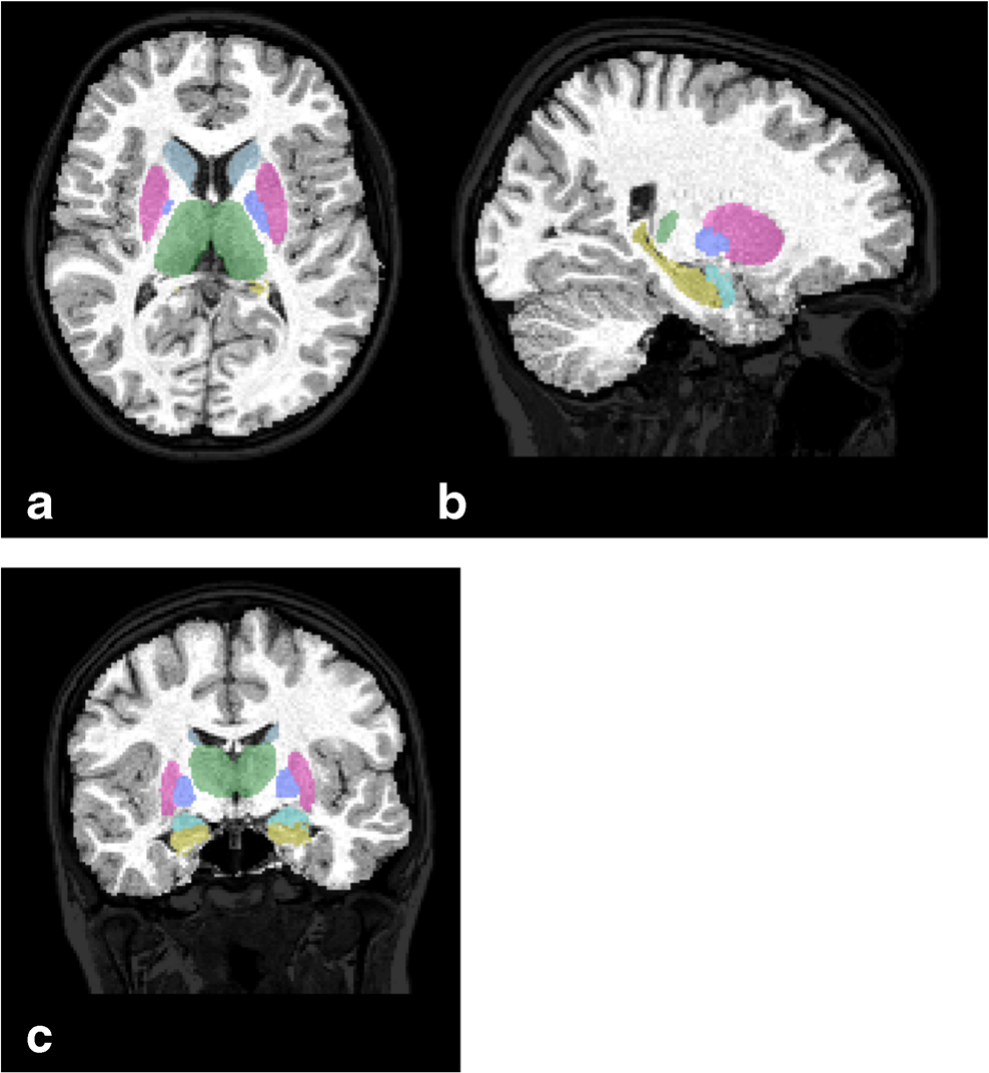
Axial (**a**), sagittal (**b**), and coronal (**c**) images of subcortical segmentation obtained using FSL-FIRST tool. Label *colors* represent the thalamus (*green*), putamen (*pink*), pallidum (*dark blue*), caudate nucleus (*light blue*), amygdala (*cyan*), and hippocampus (*yellow*)

Patients were scanned using 1.5 T Magnetom Avanto scanner (Siemens, Erlangen, Germany): standard 12-channel head matrix coil. Structural brain imaging was assessed using 3D T1-weighted magnetization-prepared rapid acquisition gradient echo sequence (TR/TE/TI = 2400/3.61/1000 ms, flip angle = 8°, voxel dimensions = 1.20 × 1.25 × 1.25 mm, Grappa = 2). Image processing was performed using FSL (Functional Magnetic Resonance Imaging of the Brain—FMRIB Software Library) 5.0-FMRIB’s Integrated Registration and Segmentation Tool (FIRST) software package to obtain the volume of total WM and GM as well as subcortical structures (thalamus, caudate, putamen, hippocampus, amygdala, globus pallidus, the whole brain) [14–16]. Following registration to a standard template, this software uses a Bayesian probabilistic model that relies on shape and intensity to infer the location of structures of interest. For each structure, a pre-defined number of modes is applied to ensure the best fit. Processing steps of FIRST included affine registration to standard template using FMRIB’s Linear Image Registration Tool (FLIRT), elastic registration to standard brain template using FNIRT, skull stripping using Brain Extraction Tool (BET), and tissue-type segmentation with FMRIB’s Automated Segmentation Tool (FAST). All files were visually inspected to ensure correct registration. A voxel count was then used to estimate volumes of structures segmented (Fig. 2). Finally, for all subcortical structures, left and right volumes were combined and mean values of both volumes were taken for further analysis.

### Neuropsychological assessment methods

All children enrolled into the study were evaluated using the battery of neuropsychological tests tailored to the age of a child for assessment of different aspects of memory, attention, concentration, processing speed (Wechsler Intelligence Test for Children, Rey Auditory Verbal Learning Test, verbal fluency test, and Benton Visual Retention Test), and executive functions (Stroop test, clock drawing test, and Wisconsin Card Sorting Test). All neuropsychological tests were administered in Polish standardized version, and Polish normative conversions were used to assess the results of studied group. Neuropsychological evaluation was scheduled on the same day, prior to magnetic resonance imaging.

Wechsler Intelligence Test for Children (WISC-R) is the most popular and the most widely used test to assess the intelligence quotient (IQ). Intelligence determines the ability to use one’s mental abilities to operate efficiently and respond to the changing demands of the environment [17]. The two basic ways of intelligence are verbal and non-verbal behavior. This was reflected in the Wechsler scale to measure intelligence in two subscales, verbal and performance, to estimate the final result full-scale IQ. The Ray Auditory Verbal Learning Test (RAVLT) is widely used and frequently translated verbal memory measure that provides scores for different aspects of memory. The RAVLT has proven useful in detecting dysfunction in different memory systems with patterns of learning and retrieval [18]. The verbal fluency test (VFT) is a short test of verbal functioning. Serious deficits in either verbal ability will manifest themselves in poor performance in the fluency tasks. Therefore, the fluency tasks can be used as an efficient screening instrument of general verbal functioning [19]. Benton Visual Retention Test (BVRT) is a tool for measuring the visual perception, visual short-term memory, and visuospatial cognition. BVRT allows differentiating the visual memory disorders from attention disorders. The Stroop test is used to assess selective attention and executive functioning. For children with poor reading skills (below 11 years old), we used Kit for Children of Stroop test with animal naming instead of reading. The clock drawing test (CDT) is a simple and effective test to include neuropsychological status. It also provides a significant advance in early detection and monitoring cognitive disorders. The CDT is a useful task to assess functioning of multiple brain regions, in cortical and subcortical areas, and the circuits that connect them. Impaired clock drawing can provide a signal of different cognitive impairment [20]. Wisconsin Card Sorting Test (WCST) is one of the most commonly used instruments for the assessment of executive function and is considered a significant measure of cognitive flexibility, attention, and executive functioning. The test assesses abstract reasoning, the subject’s ability to generate problem-solving strategies in response to changing conditions, and may be regarded, therefore, as a measure of flexibility of thought [21].

To analyze the obtained data, the neuropsychological test results were combined within domains of cognitive functioning they measure, i.e., intelligence quotient, memory and attention, processing speed, and executive functions. Intelligence quotient was based on WISC-R results. The memory and attention testing took into account the Verbal Comprehensive Index (VCI) calculated on the base of four subtests of the WISC-R scale (similarities, vocabulary, comprehension, and information); RAVLT scores measuring learning rate, retroactive, and proactive interference; and retention of information and VFT in both phonemic and semantic categories. Processing speed was evaluated by the Processing Speed Index (PSI) of WISC-R test containing results of coding and symbol search subtest and the Stroop test performance index. Finally, executive functioning was tested by CDT total score and WCST results (number of achieved categories, trials needed to achieve the first category, and percentage of correct answers).

### Statistical analysis

Statistical analysis was performed using Statistica v8.0. Neuropsychological performance was compared across subgroups using Mann-Whitney *U* test, whereas brain volumes were compared by Student’s *t* test. To assess group interactions in measured neuropsychological factors, we used Kruskal-Wallis test and for volumetric measurements—ANOVA. Uncorrected *p* values of ANOVA are reported throughout the manuscript, and Bonferroni corrections for multiple comparisons are also included where appropriate. Outliers were adjusted by the Grubbs’ test. Finally, to examine the relationship between neurocognitive performance and volumetric assessment, the linear regression analysis was performed. Statistical significance was set at *p* < 0.05.

## Results

### Demographic

The analyzed groups were comparable with regard to age (*p* = 0.480) and demographic and socio-economic status assessed by the parents’ self-assessment questionnaire.

### CNS structural magnetic resonance assessment

Both treated groups, whether chemotherapy only or in combination with CNS radiotherapy, had significantly lower volumes of caudate nucleus (*p* = 0.010 and *p* = 0.013) in comparison to control group. Moreover, the group of children who had received irradiation had smaller mean hippocampal volume than children treated without irradiation (*p* < 0.001) and control group (*p* = 0.004). The group treated with chemotherapy and radiotherapy had smaller mean volume of globus pallidus (*p* = 0.005) when compared with patients prior to therapy. There were no differences in the mean volumes of total WM, total GM, thalamus, putamen, and amygdala between studied groups (Table 2). These results were also confirmed by ANOVA analysis with correction for multiple comparisons (Table 3).

**Table 2:** Mean subcortical volumes based on FIRST segmentation (comparisons across subgroups)

Brain structure (mean volume)	I Chemotherapy group (*n* = 22)		II Chemo- and radiotherapy group (*n* = 11)		III ALL control group (*n* = 12)		I vs III (*t* test)		II vs III (*t* test)		I vs II (*t* test)	
	Volumes (mm^3^)											
	Mean	SD	Mean	SD	Mean	SD	*t*	*p*	*t*	*p*	*t*	*p*
Thalamus	7093	710	6710	279	7104	664	0.657	0.519	0.750	0.466	1.515	0.140
Hippocampus	4067	391	3417	220	4059	511	0.611	0.541	*3.321*	*0.004*	*4.954*	*<0.001*
Amygdala	1482	181	1402	137	1658	295	1.198	0.247	1.796	0.090	1.261	0.217
Caudate nucleus	3842	439	3796	357	4433	340	*2.767*	*0.010*	*2.828*	*0.013*	0.194	0.847
Putamen	5887	518	6015	500	5908	595	0.289	0.771	0.728	0.476	0.631	0.532
Globus pallidus	1728	235	1673	156	1957	146	1.818	0.082	*3.189*	*0.005*	1.041	0.306
Gray matter	658872	60581	680733	51924	668637	59581	0.386	0.703	0.398	0.696	0.814	0.424
White matter	499963	42262	497747	43763	524515	49034	1.312	0.201	1.062	0.306	0.113	0.911
Brain ventricles	7906	1421	7488	1470	9625	2599	*4.322*	*0.001*	*3.792*	*0.001*	0.788	0.437

All data given is raw scores (absolute volumes of brain structures)

Values in italics has statistical significance set at *p* < 0.05

**Table 3:** Mean subcortical volumes based on FIRST segmentation (group interactions)

Brain structure (mean volume)	Group interaction (ANOVA)		Post hoc correction (Bonferroni test)		
			I vs III	II vs III	I vs II
	*F*	*p*_uncorr_	*p*_Bon_	*p*_Bon_	*p*_Bon_
Thalamus	1.162	0.327	0.999	0.669	0.442
Hippocampus	*10.449*	*<0.001*	0.999	*<0.001*	*<0.001*
Amygdala	2.013	0.148	0.172	0.420	0.964
Caudate nucleus	*5.008*	*0.013*	*0.002*	*0.006*	0.998
Putamen	0.376	0.789	0.998	0.999	0.996
Globus pallidus	*3.546*	*0.047*	0.260	*0.006*	0.859
Gray matter	0.338	0.716	0.999	0.999	0.999
White matter	0.987	0.384	0.602	0.783	0.999
Brain ventricles	*12.126*	*<0.001*	*<0.001*	*<0.001*	0.999

*p*_*uncorr*_ *p* value for group interaction measured by ANOVA, *p*_*Bon*_ *p* value corrected for multiple comparisons by Bonferroni test

Values in italics has statistical significance set at *p* < 0.05

### Neurocognitive assessment

Comparison of the three studied groups revealed that patients treated with chemo- and CNS radiotherapy performed worse in memory and executive functions domain. Group II showed significantly worse long-term memory VCI (score 46.0+/−18.0 vs 44.0+/−9.0 and 59.0 +/−25.0; *p* = 0.047) and postponed recalling in RAVLT (*p* = 0.026) and worse visual-spatial memory (*p* = 0.005) in comparison with control group and group treated with chemotherapy alone. In addition, these patients performed significantly worse in executive functioning measured by achieved categories and percentage of correct answers in WCST (*p* = 0.011 and *p* = 0.012, respectively). Both studied groups, I and II, showed similar phonemic (8.7 and 7.7 vs 10.9) but worse semantic verbal fluency (17.5 and 15.0 vs 24.0) in contrast to the control group (*p* = 0.095 and *p* < 0.001, respectively). The group of patients who had not received CNS radiotherapy was found to have significantly worse results in interfering trial of RAVLT (*p* = 0.004) and Stroop test index (*p* < 0.001) compared with children before therapy. Furthermore, the significant difference between both treated groups (group I vs II) was found in the IQ assessment (*p* < 0.001) and the processing speed (*p* = 0.015). In addition, the Kruskal-Wallis test revealed significant interactions between groups for memory, attention, and executive functions. The summary of neuropsychological assessment results is given in Table 4.

**Table 4:** Summary of neuropsychological assessment

Domain/test	I Chemotherapy group (*n* = 22)		II Chemo- and radiotherapy group (*n* = 11)		III ALL control group (*n* = 12)		I vs III (Mann-Whitney *U* test)		II vs III (Mann-Whitney *U* test)		I vs II (Mann-Whitney *U* test)		Group interaction (Kruskal-Wallis test)	
	Median	IQR	Median	IQR	Median	IQR	*Z*	*p*	*Z*	*p*	*Z*	*p*	Chi	*p*
IQ assessment														
WISC-R	114.0	25.0	102.0	14.0	120	34.0	0.298	0.916	0.688	0.497	*2.641*	*<0.001*	0.987	0.844
Memory and attention														
VCI	44.0	9.0	46.0	18.0	59.0	25.0	1.446	0.159	*2.101*	*0.047*	1.069	0.285	2.256	0.498
RAVLT learning	7.0	3.0	6.0	3.0	8.0	1.0	1.829	0.156	1.287	0.189	0.592	0.554	5.485	0.116
RAVLT interference	11.0	3.0	8.0	2.4	9.0	1.0	*2.059*	*0.004*	0.534	0.597	*3.418*	*<0.001*	*17.589*	*<0.001*
RAVLT recall	11.0	2.0	7.0	3.0	10.0	2.0	0.894	0.377	*2.459*	*0.026*	*3.437*	*<0.001*	*7.598*	*0.017*
BVRT	7.5	3.0	6.0	2.0	8.5	1.0	1.889	0.072	*2.947*	*0.005*	1.852	0.064	6.856	0.122
VFT phonemic	8.7	1.7	7.7	4.7	10.9	3.0	*2.894*	*0.013*	*2.259*	*0.031*	1.375	0.169	5.688	0.095
VFT semantic	17.5	4.0	15.0	4.0	24.0	5.0	*2.987*	*<0.001*	*2.984*	*<0.001*	*3.684*	*<0.001*	*17.459*	*<0.001*
Processing speed														
PSI	24.0	5.0	21.0	4.0	24.0	2.0	0.784	0.624	1.008	0.584	*2.444*	*0.015*	3.468	0.415
Stroop test	2.9	0.4	3.2	1.0	2.5	0.3	*2.718*	*<0.001*	0.064	0.083	1.071	0.284	*7.895*	*0.018*
Executive functions														
CDT	3.0	1.0	2.0	1.0	3.0	0	1.110	0.267	1.671	0.094	0.897	0.369	0.001	1.000
WCST achieved cat.	4.5	1.0	3.0	2.0	5.0	1.5	1.658	0.099	*2.947*	*0.011*	*2.081*	*0.037*	*13.259*	*0.004*
WSCT trials to first cat.	16.0	11.0	19.0	12.0	12.0	4.0	1.386	0.201	1.724	0.136	0.573	0.567	4.658	0.198
WSCT percentage of correct answers	56.6	19.6	38.0	21.0	66.3	17.5	1.784	0.074	*2.891*	*0.012*	1.375	0.169	6.314	0.126

All data given in the table is scaled scores (adjusted for the children’s age)

Values in italics has statistical significance set at *p* < 0.05

### Subcortical gray matter volume and cognitive performance

Regression of memory, attention (VCI, RAVLT recalling, and BVRT), executive functions (WCST), and volumetric measurements revealed a significant relationship between auditory-verbal memory and hippocampus volume regardless of applied therapy (Table 5). The other neurocognitive functions analyzed were not associated with brain volumes.

**Table 5:** Linear model regression analysis of selected neuropsychological domains as a function of hippocampus volume

Cognitive domain	Beta coefficients	*p*
Memory and attention		
VCI	0.218	0.186
RAVLT recall	0.614	*0.004*
BVRT	0.168	0.279
Executive functions		
WCST achieved cat.	0.063	0.714

Values in italics has statistical significance set at *p* < 0.05

## Discussion

Our study has shown that all treated patients have decreased cognitive performance in comparison to ALL patients prior to treatment. They also have decreased hippocampal and caudate nucleus volumes. The therapeutic regimen of childhood ALL assumes diverse CNS involvement prophylaxis depending on risk group, i.e., with or without radiotherapy. Results of the study demonstrated that all included patients treated for ALL, regardless of whether or not irradiated, in contrast to some other reports and despite expectations of the ALLIC 2002 protocol users, do have cognitive functioning impairment and decreased volume of subcortical GM in hippocampus and caudate nucleus in long-term follow-up. Irradiated patients performed worse in both long-term and short-term memory, attention, and executive tasks.

In the study, patients treated with CNS irradiations were proven to have smaller hippocampus volumes in comparison with children treated with chemotherapy only and the control group. The main reason for selecting hippocampal volume as imaging outcome was its crucial role for memory processing and encoding declarative memories. The domains of radiation-related neurocognitive decline encompass immediate and delayed verbal memory with or without non-verbal memory [22]. However, Sun et al. [23] reported that even though verbal memory was likely to deteriorate significantly, general non-specific cognitive functions were not influenced. Our results provided the same conclusions that only selected cognitive functions were significantly affected in ALL survivors, while IQ levels were comparable with IQ levels of the control group. As given in the literature, in our study, the most affected cognitive domains were those related to the hippocampal area, i.e., immediate and delayed verbal memory.

The mechanism of cognitive decline after irradiation of hippocampal area remains unclear. It is well known that the hippocampi are extremely vulnerable to chemotherapy and radiotherapy [24–27]. Injury of neuronal stem cells located in the hippocampal region might contribute to long-term impact on cognitive processing. The cognitive impairment after radiotherapy results from inhibition of hippocampal neurogenesis. Cheng et al. [28] suggest that despite aging-associated decline, the neurogenesis may be further inhibited by ablation of neural progenitors and activation of microglia in hippocampus caused by irradiation. This may explain the results of our study and correlation between hippocampus injury and memory deficits in patients who were irradiated. These deficits were not found in children who did not receive cranial irradiation.

We also found a significant reduction of caudate volume in both groups of ALL survivors treated with or without irradiation. The caudate, as part of the striatum, is a part of the frontostriatal circuit supporting executive functions [29], and caudate lesions have been shown to lead to impaired processing speed, attention, learning, memory, and verbal fluency [30, 31]. Remarkably, these functions were also found to be affected in our cohort.

Globus pallidus is typically associated with motor deficits, including Parkinsonism, tremor, and dystonia. However, less commonly known, this area may also be associated with cognitive functioning. Cognitive domains related to pallidum area include executive functions and verbal memory [32]. Dysfunction of globus pallidus results in worsening general cognition, immediate recall problems, and deterioration of executive functions [33]. In our study, the worse executive functions performance found in irradiated ALL survivors may be related to smaller pallidum volume in this group of patients.

The literature data on WM and GM volume changes after childhood ALL treatment remains unclear. In our study, we found no statistical differences in total WM and GM volumes between both studied groups and the control group. However, there was a trend to smaller WM volume in both treated groups. Other authors also reported smaller WM volumes in ALL survivors [34–36]. Reddick et al. [34] have previously attempted to address the brain volume reduction as a result of ALL therapy, and they have found smaller WM volumes and no differences in GM volumes, even in children receiving 18 Gy when compared to healthy controls. However, Reddick et al’s structural analysis did not assess subcortical structures, but only total GM. Similarly, Kesler et al. [35] found no difference in total brain volume but significantly reduced regional WM volumes in ALL survivors compared with the controls. Finally, Carey et al. [36] described two specific regions of reduced WM in the right frontal lobe in ALL survivors compared with healthy controls. In previous studies of ALL patients, there was little focus on regional GM, especially on subcortical structures. Porto et al. [37] reported reduced GM concentration within the caudate and thalamus in irradiated survivors of childhood ALL, while Zeller et al. [38] found that ALL survivors had significantly smaller volumes of cortical GM, amygdala, caudate, hippocampus, and thalamus compared with healthy controls. In the study, we showed smaller volume of hippocampus, caudate, and globus pallidus in irradiated ALL survivors with no difference in total GM volumes. The reason for these differences between studies may be related to technical difficulties segmenting small structures such as the hippocampus. Moreover, between these studies, there are imaging parameter differences, i.e., other scanners’ parameters and acquisition time.

The mechanism of neurotoxic consequences of ALL therapy is complex. There are two explanations considering decreased volumes of brain. One mechanism is damage to oligodendroglial cells resulting in demyelination and another atypical WM development [39]. The other possible mechanism is vascular damage. Methotrexate inhibits enzymes involved in folic acid biosynthesis. Decreased folate causes elevation of homocysteine, an amino acid toxic for blood vessel endothelium. The final result is microangiopathy of small blood vessels with its occlusion, particularly vulnerable in the basal ganglia vessels [40, 41]. Selective vulnerability of neuronal and glial cell populations implicates that certain subgroups of cells will die shortly following therapy, and remaining intact cells will reorganize cytoarchitectonic structure of both GM and WM. However, GM reorganization might also play a more important role in affecting developmental trajectory, as well as WM role that is well proven.

In our study, the children treated with chemotherapy alone were also found to have cognitive deficits. Even though they achieved results comparable to control group in the intelligence test, long-term and short-term memory domain, they had significantly lower processing speed than the control group. However, previous papers usually connected slowed profile information processing with a consequence of radiotherapy; it is uncertain if chemotherapy alone may also cause this cognitive dysfunction [42–47]. Possible explanation given in the literature is decreased cerebral volume, which is associated with poorer cognitive outcome, especially slow processing, and attention problems [48]. Although WM loss is greater in children treated with radiotherapy, its volumes in ALL survivors treated with chemotherapy are decreased only in relation to the control groups in many reports [34, 44, 49, 50]. In our study, there was also the trend to smaller volumes in both treated groups when compared to controls, but due to the small sample size, it was not statistically confirmed.

Executive function performance was significantly worse in children who had previously received irradiation in contrast to children treated with chemotherapy alone. Other results were found in Buizer et al.’s [51, 52] studies, who found that executive function deficits are a main feature in ALL survivors treated with chemotherapy alone. The explanation may be the higher dose of chemotherapy (MTX) in the group treated with irradiation than in the group treated with chemotherapy alone in our study. The cumulative dose of intravenous methotrexate correlates with the severity of deficits. Executive functioning is based on networks localized in cerebellar prefrontal area. The myelination of the prefrontal cortex has a protracted course during childhood and adolescence [26, 53]. The less mature brain seems to be more vulnerable to chemotherapy-induced damage than the more mature areas. Thus, prefrontal cortex defects and its functional implications, i.e., executive function deficits, are observed in all children treated due to ALL, even in those treated without irradiation [53].

Our study is limited due to a small number of patients and cross-sectional design. Further multi-center studies on larger groups of patients are necessary to confirm our preliminary results. Another limitation is the cross-sectional design of the study and lack of information on structural brain changes occurring prior to diagnosis and at the time of the treatment initiation. Furthermore, the structural imaging had relatively large voxels which would result in partial voluming in small convoluted structures, such as hippocampus. Nevertheless, we propose that detailed structural assessment of regional brain volume is possible in clinical settings provided that an MRI study will include a volumetric sequence that enables more detailed analysis like the one used in this study.

Our results are preliminary and only the first step to assess long-term side effects of high-dose methotrexate administered alone or in combination with cranial irradiation in dose reduced to 12 Gy as a CNS prophylaxis in ALL children. Further studies should also focus on finding the mechanism of brain damage by examining the functional connectivity and structural features of the neuronal pathways.

In summary, our results provide the first assessment of long-term brain structural magnetic resonance imaging results and neurocognitive functioning of children treated according to ALLIC 2002. These late effects are even more pronounced in children treated with CNS radiotherapy. It seems to be essential to further reduce the role of irradiation as part of CNS prophylaxis in children treated for ALL. Further studies based on a larger group of patients are necessary to confirm these observations.
